# Generation of triacyl lipopeptide-modified glycoproteins by metabolic glycoengineering as the neoantigen to boost anti-tumor immune response

**DOI:** 10.7150/thno.60211

**Published:** 2021-05-25

**Authors:** Yujia Zhao, Siyu Li, Jianying Lv, Yonghui Liu, Yanan Chen, Yanhua Liu, Xiaosu Chen, Jia Li, Xuan Qin, Xiaoshuang Wang, Jie Shi, Yi Shi, Rong Xiang

**Affiliations:** The school of Medicine, Nankai University, Tianjin 300071, China.

**Keywords:** tumor immunotherapy, glycoengineering, tetra acetyl-N-azidoacetyl-mannosamine, Pam3CSK4, PD1/PD-L1

## Abstract

The lack of tumor specific antigens (TSA) and the immune tolerance are two major obstacles for the immunotherapy of cancer. Current immune checkpoint inhibitors (ICIs) show clinical responses in only limited subsets of cancer patients, which, to some extent, depends on the mutation load of tumor cells that may generate neoantigens. Here, we aimed to generate a neoantigen MDP to exhibit stronger anti-tumor efficacy.

**Methods:** In this study, we utilized chemically modified sialic acid precursor tetra acetyl-N-azidoacetyl-mannosamine (AC_4_ManNA_Z_) to engineer the glycoproteins on the membranes of tumor cells for the covalent ligation of hapten adjuvant Pam3CSK4 *in vivo*, which eventually generated a neoantigen, i.e., ManNA_Z_-DBCO-Pam3CSK4 (MDP), on tumor cells. The high labeling efficiency, relatively specific biodistribution in tumor tissues and the anti-tumor efficacy were confirmed in the syngeneic murine models of the breast cancer and the lung cancer.

**Results:** The generation of MDP neoantigen in tumor-bearing mice significantly evoked both the humoral and the T-cell-dependent antitumor immune responses, resulting in a strong inhibition on the growth of the breast cancer and the lung cancer allografts and significantly prolonged survival of tumor-bearing mice. Interestingly, MDP neoantigen was able to dramatically increase the sensitivity of cancer cells to ICIs and greatly enhance the anti-tumor efficacy in the murine models of both breast cancer and the lung cancer, which showed no or low responses to the immunotherapy with anti-PD1 antibody alone.

**Conclusions:** We developed a simple metabolic glycoengineering method to artificially generate neoantigens on tumor cells to enhance tumor cell immunogenicity, which is able to significantly improve the response and the clinical outcome of ICIs.

## Introduction

The lack of tumor-specific antigens and the activation of immune suppressive mechanisms are major reasons why tumor cells can escape from the immune surveillance, which also become the biggest challenges for tumor immunotherapy 1. Big progress has been made in tumor immunotherapy recently by the utilization of immune checkpoint inhibitors (ICIs), including anti-PD-L1/PD-1 2-4 and anti-CTLA-4 5-7 antibodies, to overcome the tumor immunosuppressive environment, which shows clinical efficacy in the treatment of various cancers including melanoma, ovarian cancer, colorectal cancer and lung cancer 8, 9. However, many cancer patients show no or very low responses to ICIs, which seems not to depend on the expression of tumor self-antigens 10, but correlates with tumor mutation burden (TMB) that may generate tumor neoantigen 11-13, highlighting the value of the generation of cancer neoantigens in improving the efficacy of ICIs.

The aberrant expression of glycosyltransferases and glycosidases leads to the abnormal glycosylation of the glycoproteins or glycolipids on the surface of the tumor cells 14, resulting in the generation of abundant tumor-associated carbohydrate antigens (TACAs) 15, which is one of the distinctive features of various malignant tumors 16, 17. Significantly increased sialylation on the N-linked and O-linked oligosaccharide chains on the cell surface glycoprotein has been widely found in cancer cells 18, which contributes to tumor progression and poor prognosis 16. Therefore, sialic acid has been utilized as an ideal target for developing tumor vaccines 15. However, the natural TACAs are reported to mainly activate B lineage cells to transiently produce IgM antibodies with low affinity due to the lack of pattern molecules, while the T-cell-dependent immune responses are seldomly activated by TACAs 15, resulting in the low efficacy in treating tumors 19. To activate T-cell-dependent immune responses, many immune adjuvants containing different pattern molecules were developed to activate dendritic cells (DC) by interacting with the pattern recognition receptors (PRRs) of DC and hence the activation of the innate immune system 20, 21. Toll-like receptor 2 and 3 (TLR2/3), which abundantly expressed on human and murine DCs 22, are PRRs that recognize lipopolysaccharide and lipopeptide, and agonists of them are widely used as adjuvants in cancer vaccines. TLR2/3 can activate the pro-inflammatory NF-κB, promote the antigen presentation, stimulate the production of inflammatory cytokines, such as IL-8, IL-12 and TNF-α, and eventually promote the maturation of naive T lymphocytes 23.

Currently, adjuvants can be conjugated to cell surface glycoproteins through click chemistry-based glycoengineering. The click reaction can occur under physiological conditions with high stereospecificity, high yield and few cytotoxic by-products 24. Among the classic click chemistry reactions, copper-free catalyzed strain-promoted azide-alkyne cycloaddition (SPAAC) reaction of dibenzyl cyclooctyne (DBCO) and azide is widely used due to its fast reaction rate under the physiological condition and lower toxicity 25, 26. Tetra acetyl-N-azidoacetylmannosamine (AC_4_ManNA_Z_) has been used to introduce azido-group in living cells which can be metabolized to N-azido acetylneuraminic acid and eventually incorporated into the glycoproteins and glycolipids on the cell surface 27-29, while exhibiting very low toxicity 30.

Here, we utilized the metabolic glycoengineering method to covalently add the immune adjuvant on the surface of tumor cells to generate tumor neoantigen *de novo* in mice, aiming to activate the anti-tumor immune responses. We selected the triacyl lipopeptides Pam3CSK4 (Pam3Cys-Ser-(Lys)4), a derivative of bacterial lipopolysaccharide (LPS), as the adjuvant, which was covalently linked to the sialic acid of the glycoproteins or glycolipids by the click reaction in tumor cells fed with chemically modified sialic acid precursor AC_4_ManNA_Z_ to generate an artificial tumor neoantigen, namely MDP. In the murine models of both breast cancer and NSCLC, the *de novo* synthesis of MDP neoantigen can significantly enhance the anti-tumor immune responses and inhibit the growth of breast cancer and lung cancer homografts. Interestingly, for the cancer types that show low response to ICIs, the combination of MDP neoantigen dramatically enhance the sensitivity to ICIs and exhibit excellent anti-tumor efficacy.

## Methods

### Cells and mice

The murine breast cancer cells 4T1-luciferase and the murine Lewis lung carcinoma cells LLC-luciferase were cultured in Dulbecco's modified Eagle's medium (DMEM; Biological Industries (BI)) supplemented with 10% fetal bovine serum (FBS; BI), 100 U/mL of penicillin and 100 μg/mL of streptomycin (BI).

The six to eight weeks old female BALB/c mice and C57BL/6 mice used in the mouse experiments were purchased from Sibeifu Experimental Animal Technology Co. Ltd. (Beijing, China) and allowed to acclimate for one week before use. All mice were maintained in a pathogen-free animal facility with a 12 h light/dark cycle. All murine care and experiments were performed according to the guidelines approved by the Animal Care and Use Committee at Nankai University (Tianjin, China).

### The murine models of the breast cancer and NSCLC allografts

For the syngeneic murine model of breast cancer, 4T1-luciferase cells (4×10^5^) were suspended in 100 μL PBS and injected subcutaneously between the second and third pairs of mammary glands near the right armpit of BALB/c mice. For the syngeneic murine model of NSCLC, LLC-luciferase cells (1×10^6^) were suspended in 100 μL of solution containing equal volumes (i.e., 50 μL) of medium and Matrigel (BD Biosciences) on ice and injected into the lung at the midpoint between the prosternum and right armpit on the right lung of C57BL/6 mice. When tumors were palpable or bioluminescence signals were visible, mice were randomly grouped (8 mice/per group) and received intravenous administration of 40 mg/kg AC_4_ManNA_Z_ (Kaisenlai technology Co., Ltd., Beijing, China), Mannose (as a control group) or PBS (as blank control group) for 3 consecutive days. In the tracing experiments, 5 mg/kg of DBCO- Cy5 (Sigma-Aldrich, St Louis, MO, USA) or DBCO-Pam3CSK4-Rhodamine or PBS was injected via the tail vein on the fourth day right after the administration of AC_4_ManNA_Z_. For the generation of MDP neoantigen *in vivo*, 5 mg/kg of DBCO-Pam3CSK4 (DP for short hereafter), which was estimated to be 4×10^16^ molecules of DP-conjugated glycoproteins, or PBS was injected via the tail vein on the fourth day right after the administration of AC_4_ManNA_Z_ and this injection procedure was repeated every week. For the treatment with anti-PD1 antibody, 10 mg/kg of mouse anti-PD1 antibody or mouse anti-IgG-2a antibody (BioXcell, West Lebanon, NH, USA) dissolved in 100 μL PBS was injected intraperitoneally at the same timepoint when DP was administrated.

For the tracing experiment, 48 h after the tail vein injection of 5 mg/kg of DBCO-Cy5, the mice were sacrificed and the vital organs (heart, liver, spleen, lung, kidney, and Brain) and tumor tissues were dissected and placed in a 10-cm Petri dish, the fluorescent signal of Cy5 was analyzed by the Caliper Life Science IVIS Lumina II Imager (Waltham, MA, USA) with fluorescent luminescence imaging mode. Tumor volume (V) was measured by vernier calipers twice a week and calculated by the standard formula: V = length × width2/2 since the 7^th^ day after inoculation. We also use the Caliper Life Science IVIS Lumina II Imager to monitor the bioluminescence signal weekly, which was analyzed by the Living Image software (Xenogen, Alameda, California, USA). For the survival analysis of 4T1 breast cancer-bearing mice, the endpoint was set to the time point when the size of the tumor allograft reached 1,000 mm^3^. For the survival analysis of LLC allograft-bearing mice, the end point was the time point when all mice in the control group died.

### DBCO-adjuvant tracing *in vivo* and *in vitro*

#### *In vitro* detection of the azide group

The 4T1 cells (5×10^4^) were seeded on the coverslips placed in a 24-well plate 24 h before 50 μM of AC_4_ManNA_Z_ was added and incubated for 72 h at 37 °C. The same volume of PBS was added as a control group. The cells were then cultured in the medium containing 50 μM of DBCO-Cy5 for 1 h at 37 °C. After fixed with 4% paraformaldehyde, Phalloidin (2.5 μg/mL) and DAPI (1 μg/mL) were added to stain the actin filaments and the nuclei. Photographs were captured by a FV-1000 confocal microscope (Olympus) with the 100× oil lens.

#### *In vivo* detection of the azide group

The vital organs and tumor tissues were dissected and embedded in the OCT compound and subjected to frozen section. The sections were blocked with 5% bovine serum albumin (BSA) for 2 h, and then incubated with 20 mM of DBCO-Cy5 for 30 min. Photographs were taken by a FV-1000 confocal microscope (Olympus) with the 100× oil lens.

#### Detection of azido-modified glycoproteins

Azido-modified glycoproteins in the vital organs and tumor tissues were extracted with RIPA lysis buffer (50 mM Tris-HCl pH 7.4, 150 mM NaCl, 1% NP-40, 0.1% SDS). 20 μL of proteins (5 mg/mL stock solution) were incubated with 2 μL of 5 mM EZ-Link^®^ Phosphine-PEG_3_-Biotin (PBS dissolved, Thermo Fisher Scientic, Waltham, MA, USA) at 37 °C for 2-4 h or at room temperature (RT) for 16-24 h. After heating at 95 °C for 10 min, the proteins were resolved by SDS-PAGE and transferred to the PVDF membrane, which was blocked in 5% BSA for 1 h at RT and incubated with HRP-Streptavidin Conjugate (1:2000, TBST diluted, Thermo Fisher Scientic, Waltham, MA, USA) at 4 °C overnight. The azido-modified proteins were detected by a chemiluminescent ECL kit (Thermo Scientific, Waltham, MA, USA).

### Quantitative real time RT-PCR (qRT-PCR)

RT-qPCR was performed by using the Hieff^TM^ qPCR SYBR^®^ Green Master Mix kit (Yeasen Biotech, Shanghai, China) on Light Cycler 96 (Roche, Basel, Switzerland). The 2-ΔΔCt method was used to do the relative quantification. The primer sequences were listed in **Table S1.**

### Hematoxylin and eosin (H&E) staining

Paraffin sections of tumors and vital organs were embedded in paraffin to prepare paraffin sections. Slides of 5 μm in thickness were stained with hematoxylin and eosin (H&E) for the pathologic analysis.

### Immunohistochemistry staining (IHC)

The sections were incubated overnight at 4 °C with the primary antibody against CD8 (1:200, Abcam ab22378, Cambridge, MA, USA). The infiltration of CD8+ T cells in the tumor was quantified by counting the proportion of CD8 positive cells and the intensity of staining in each microscopic field, which was then calculated by H-score method, i.e., H score = P (positively stained area) × I (staining intensity). P value was defined as 0-4 points: 0 for < 5%, 1 for 5% ~ 25%, 2 for 25% ~ 50%, 3 for 50% ~ 75% and 4 for 75% ~ 100%. I value is defined as 0-3 points: 0 for none, 1 for weak, 2 for medium and 3 for strong.

### Immunofluorescent staining

Tumor tissues and vital organs were fast frozen after embedded with OCT compound to prepare frozen sections. Slides of 5 μm in thickness were fixed with precooled anhydrous methanol at -20 °C for 15 min and then blocked in 2% BSA for 2 h at RT. The frozen sections were incubated with the primary antibody against PD-L1/CD274 (1:50, Proteintech 17952-1-AP, Chicago, IL, USA) or CD8 (1:200, Abcam ab22378, Cambridge, MA, USA) at 4 °C overnight. Subsequently, the slides were incubated with goat-anti-rabbit conjugated Alexa 594 or goat-anti-rat antibody conjugated Alexa 488 (ZSGB-BIO, Beijing, China) for 1 h at RT in the dark. For nuclei staining experiment, frozen sections were stained with DAPI (1:1000) for 1 min at RT. Photographs were captured by a FV-1000 confocal microscope (Olympus) with the 100× oil lens.

### Enzyme-linked immunosorbent assay (ELISA) and LDH assay

The ELISA and LDH assay were conducted in accordance with the manufacturers' protocols of Dldevelop (Wuxi, China) and Thermo Fisher Scientic (Waltham, MA, USA), respectively.

### Flow cytometry analysis

EDTA were used to suspend the MDP modified cancer cells for 10-15 min. After centrifugation, the cells were fixed with 4% paraformaldehyde for 10 min and resuspended to a density of 107/mL. Subsequently, the cells were transferred to 1.5 mL tubes (100 μL/each) and incubated with serum (2 μL/each) from different groups of mice for 30 min on ice. After washing, the cells were incubated with fluorescent secondary antibody (0.5 μL/each) for 20 min on ice. For the analysis of T cell proportion in spleen, the gently grinded lymphocytes were treated with 5 mL erythrocyte lysate to lyse the red blood cells and incubated with primary antibody against CD3-FITC, CD44-PE, CD62L-APC (1:1000, Biolegend, San Diego, CA, USA), CD8-PE/CY7 (1:1000, ebioscience, San Diego, CA, USA) for 0.5-1 h at RT or overnight at 4 °C. The cells were then resuspended in PBS with 1% FBS and analyzed using a digital BD Caliber flow cytometer (BD Biosciences, San Jose, CA, USA) immediately. Data were analyzed by FlowJo software.

### Statistical analysis

The statistical analysis was performed by using GraphPad Prism 7. An unpaired Student's t-test was used for the comparisons between two different groups. A log-rank test was used for the comparisons of survival curves. Statistical result is indicated as follows: *P < 0.05, ** P < 0.01, *** P < 0.001. The P value less than 0.05 was considered statistically significant.

## Results

### Design and synthesis of MDP by metabolic cancer cell labeling

**Figure 1:**
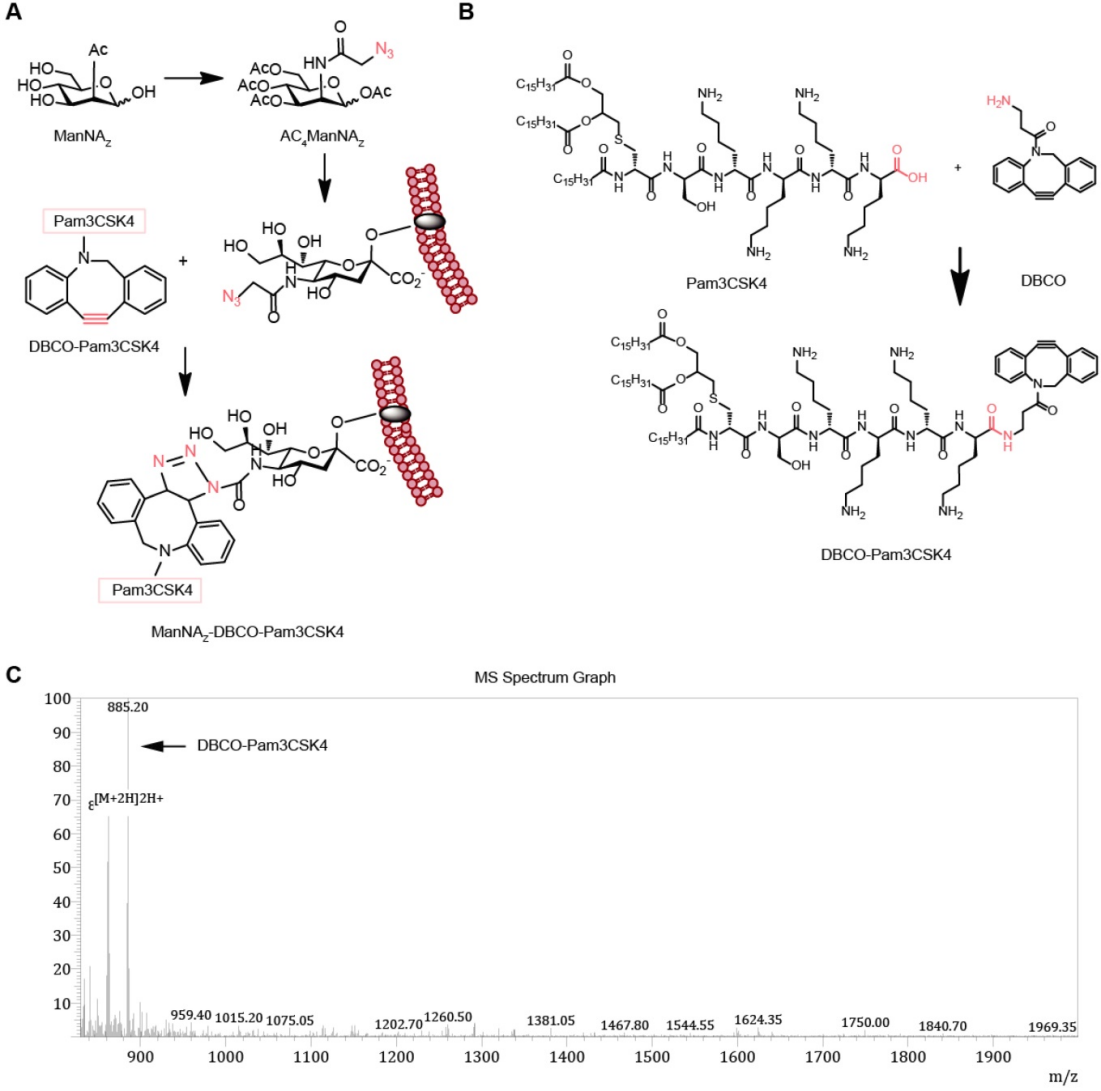
** Design and synthesis of MDP neoantigen on the surface of cancer cells. (A)** Schematic diagram of the metabolic incorporation of AC_4_ManNA_Z_ in cancer cells and the subsequent linkage of DP with azido groups via catalyst-free click chemical reaction. **(B)** Synthetic route of DP. **(C)** Characterization of DP by LC-MS analysis. LC-MS (m/z) calculated for C_99_H_170_N_12_O_13_S 1768.65, found 1768.40 [M+2H]2H^+^, 30% yield and 90% purity.

To enhance the activity of synthetic carbohydrate antigens in boosting the anti-tumor responses, we selected Pam3CSK4, which is an effective TLR2 agonist that activates the innate immune system, as the pattern molecule to add to the carbohydrate antigens. We fed the cells with the synthetic carbohydrate AC_4_ManNA_Z_, an analog of the natural sialic acid precursor N-Acetylmannosamine (ManNAc) which was able to be utilized for the synthesis of sialoglycans with corresponding sialic acid analog and presented on the cell surface as parts of the glycoproteins (**Figure 1A**). The azide group on the sialic acid analog allows the covalent linkage of the (DBCO)-modified Pam3CSK4 by click chemistry reaction without the need of any catalyst to form a neo-tumor antigen complex MDP on the surface of tumor cell (**Figure 1A-B**). The synthesis of DP (DP for short hereafter) was verified by mass spectrometry (**Figure 1B**) and there was no significant cytotoxicity (**Figure S1**).

### AC_4_ManNA_Z_ mediates selective labeling of tumor cells with DBCO-conjugated adjuvants *in vitro* and *in vivo*

**Figure 2:**
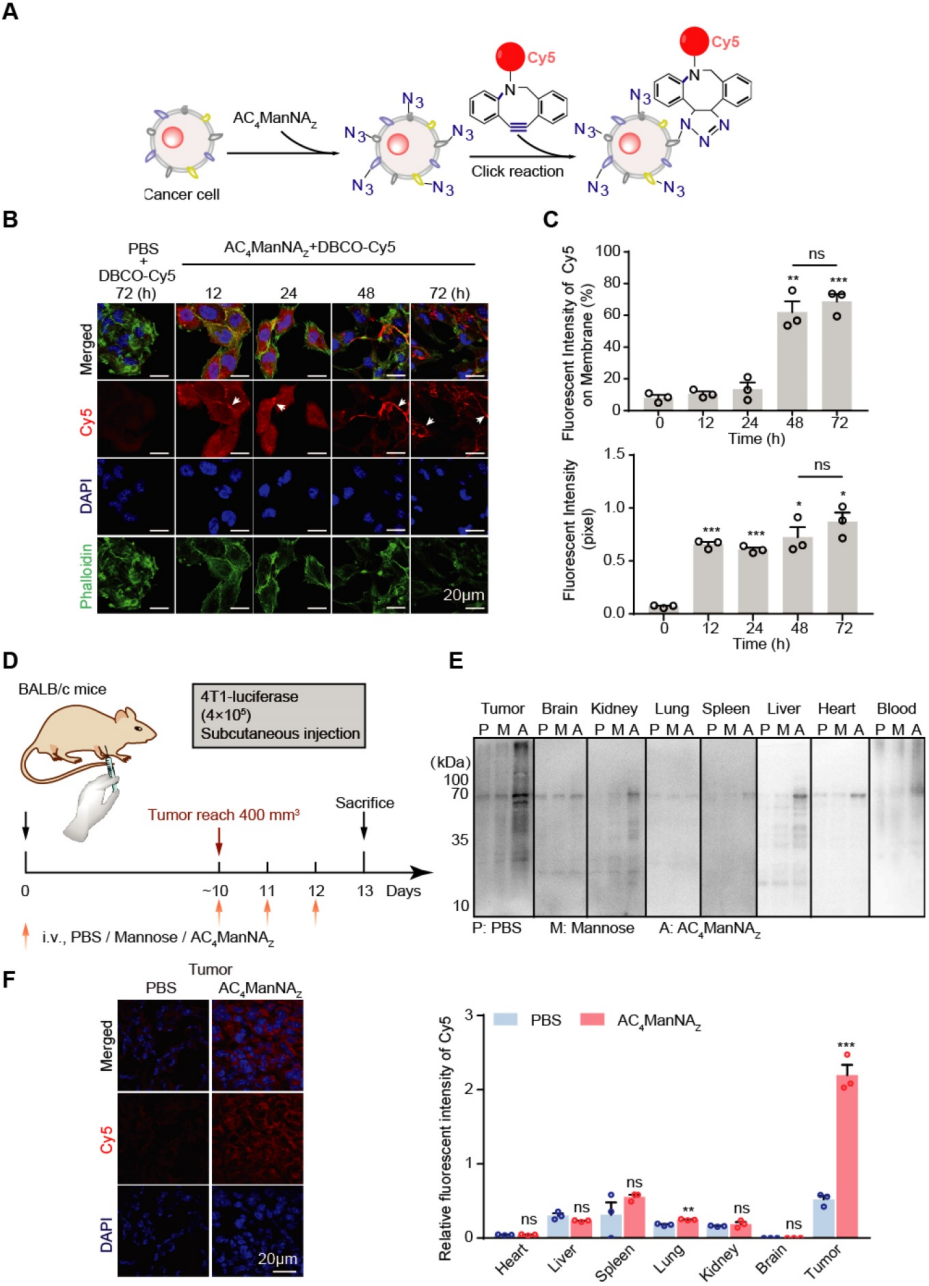
**Incorporation of AC_4_ManNA_Z_ in breast cancer cells. (A)** Schematic diagram of tracking the metabolic incorporation of AC_4_ManNA_Z_ in cancer cells using DBCO-Cy5 via click chemical reaction. **(B)** Representative images of DBCO-Cy5 in 4T1 cells after indicated treatment of AC_4_ManNA_Z_ (50 μM) and DBCO-Cy5 (50 μM, 1 h) **(C)** Quantification of the Cy5 signals in **(B)**. Data are shown as means±SD from three independent experiments. **(D)** Schematic diagram of tracking AC_4_ManNA_Z_ incorporation in a murine breast cancer model. When the tumors were palpable, mice were randomly grouped and received intravenous administration of AC_4_ManNA_Z_ (40 mg/kg), Mannose (40 mg/kg) or PBS for 3 consecutive days. Mice were sacrificed on the fourth day right after the last administration, tumors and vital organs were dissected to detect the expression of azido groups by western blot and immunofluorescence staining analysis. (**E**) Western blot analysis of the azide-modified proteins in different tissues. (**F**) Representative images of the DBCO-Cy5 signals in the tumor allografts (left panel) and the quantification (right panel). Data are shown as means±SEM (n = 3 mice, ns not significant, *P < 0.05, **P < 0.01, ***P < 0.001 by unpaired Student's t-test).

To monitor the metabolic labeling of cancer cells with MDP neoantigen on the cell surface, we firstly used the fluorescent dye Cy5-conjugated DBCO (DBCO-Cy5) to trace the efficiency and the subcellular location of the AC_4_ManNA_Z_-mediated labeling in tumor cells (**Figure 2A**). As expected, murine breast cancer cells 4T1 cultured with AC_4_ManNA_Z_ before the addition of DBCO-Cy5 showed fluorescent signals on the cell surface in a time-dependent manner (**Figure 2B-C**). In the first 24 h, the fluorescent signals reached the highest level and distributed everywhere in the cells. After 24 h, the labeling signals were mainly confined to the cell surface, indicating the successful metabolic labeling of cell surface glycoproteins (**Figure 2B-C**). In the absence of AC_4_ManNA_Z_, DBCO-Cy5 alone showed almost no specific staining of the cells, suggesting the labeling depends on the metabolic introduction of AC_4_ManNA_Z_.

Next, we investigated the metabolic labeling in a syngeneic murine model of the breast cancer (**Figure 2D**). In the Balb/c mice bearing 4T1 tumor allografts, AC_4_ManNA_Z_ was injected through tail vein once daily for 3 days. The azide-conjugated proteins from different tissues were analyzed by EZ-Link^®^ Phosphine-PEG_3_-Biotin and HRP-Streptavidin Conjugate as described in the methods above 27. Tail vein-injected AC_4_ManNA_Z_ was efficiently uptaked by tumor tissues, with much less incorporation into the other vital organs (**Figure 2E**), which was further confirmed by labeling the tissue sections with DBCO-Cy5 through *in vitro* click reaction (**Figure 2F** and **Figure S2**).

**Figure 3:**
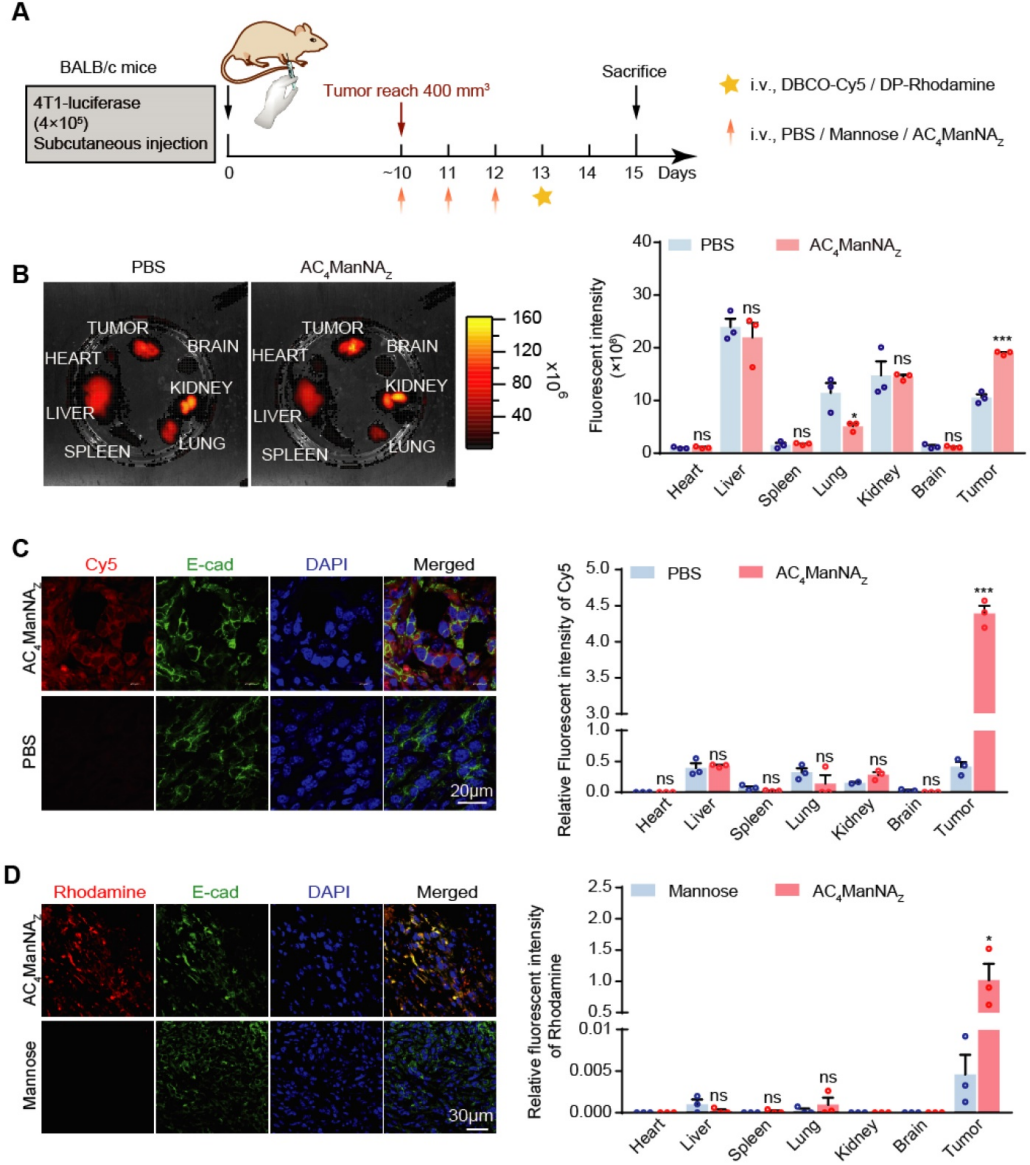
** Detection of the biodistribution and labeling efficiency of MDP *in vivo*. (A)** Schematic diagram of the mouse experiment. When the tumors were palpable, mice were randomly grouped and received intravenous administration of AC_4_ManNA_Z_ (40 mg/kg), Mannose (40 mg/kg) or PBS for 3 consecutive days. Mice were sacrificed at 48 h after labeled by DBCO-Cy5 (5 mg/kg) or DBCO-Pam3CSK4-Rhodamine (5 mg/kg), tumors and vital organs were dissected to detect the biodistribution and the labeling efficiency of DBCO-cargo by fluorescent luminescence and immunofluorescence staining analysis** (B)** IVIS fluorescence images of tumor tissues and vital organs after cardiac perfusion by the ligation of DBCO-Cy5 via click chemical reaction (left panel) and the quantification (right panel). **(C)** Representative fluorescent images of the tumor sections stained by DBCO-Cy5 ligation (left panel) and the quantification of the signals in major organs and tumors (right panel). **(D)** Representative images of the tumor sections with the modification of DP-Rhodamine (left panel) and the quantification of the signals in major organs and tumors (right panel). Data are shown as means±SEM (n = 3 mice, ns not significant, *P < 0.05, **P < 0.01, ***P < 0.001 by unpaired Student's t-test).

To further investigate the linkage of DBCO-conjugated adjuvants with azide group-modified glycoproteins *in vivo*, the BALB/c mice bearing 4T1 tumor allografts was injected through tail vein with AC_4_ManNA_Z_ once daily for 3 days, followed by the injection of DBCO-Cy5 and fluorescent signal examination (**Figure 3A**). Mice pre-treated with phosphate saline buffer (PBS) were used to monitor the noncovalently linked DBCO-Cy5 in different organs. The fluorescence images of major organs showed that the DBCO-Cy5 was mainly linked to the tumor allografts and the liver, while the other organs showed very low level of covalently-linked DBCO-Cy5 (**Figure 3B**). And the tissue sections showed more clearly that DBCO-Cy5 was mainly conjugated to the surface of tumor cells, while very little DBCO-Cy5 was found in the sections of other major organs (**Figure 3C** and **Figure S3A**).

We further monitored the *in vivo* labeling of tumor cells with DP by giving the mice Rhodamine-conjugated DP, i.e., DP-Rhodamine, after the injection of AC_4_ManNA_Z_ (**Figure 3A**). Interestingly, we observed even more specific labeling of tumor cells with DP (**Figure 3D** and **Figure S3B**), which might be due to the large size of Pam3CSK4 that greatly reduces its uptake by the other cells. These results demonstrated that *in vivo* glycoengineering by using AC4ManNAZ can efficiently and specifically link DBCO-conjugated adjuvants to the surface of tumor cells.

### Generation of MDP neoantigen on tumor cells suppresses the growth of breast tumor allografts and improves the survival of tumor-bearing mice

**Figure 4:**
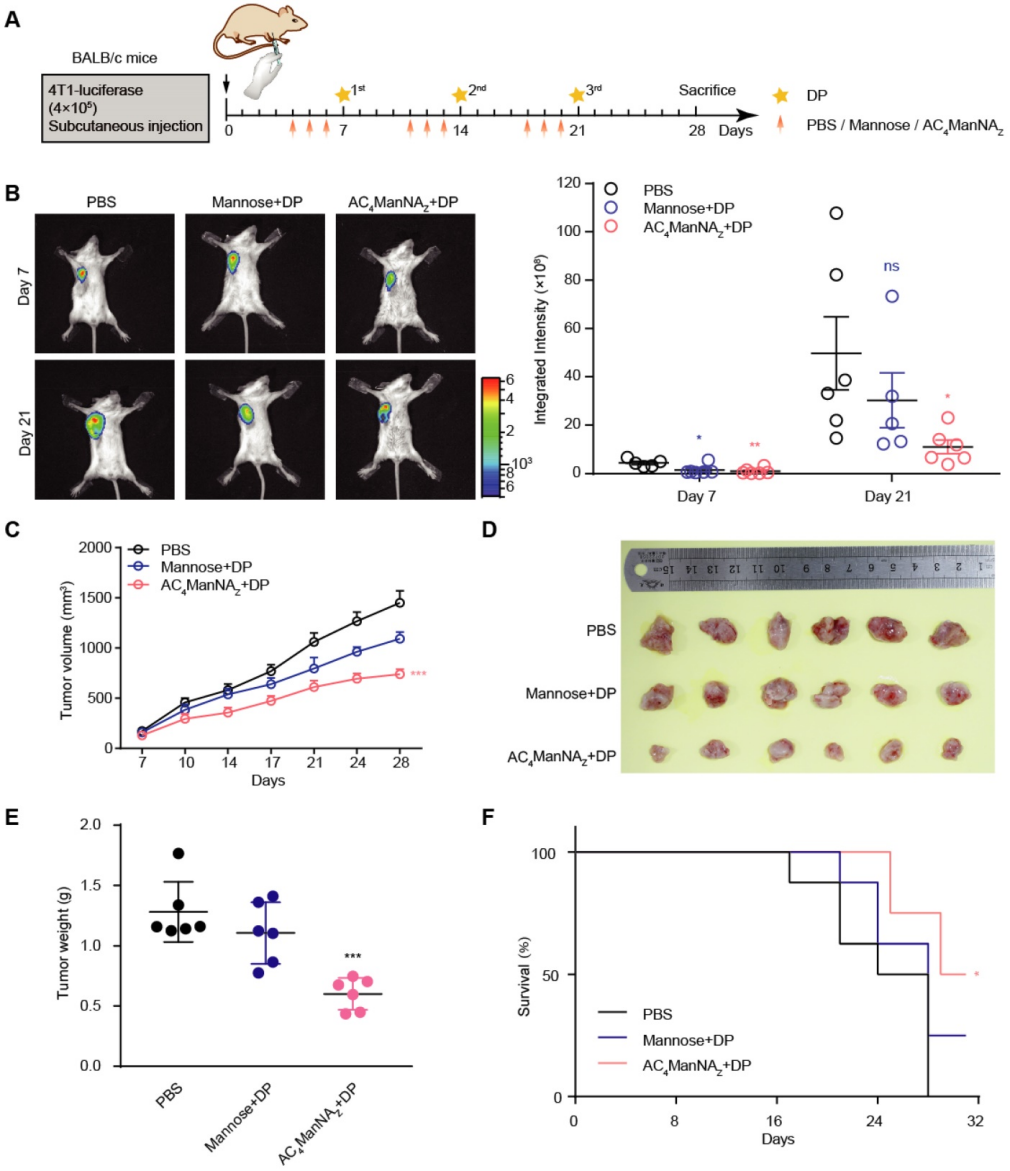
** The *in vivo* generation of MDP neoantigen exhibited antitumor effects in the breast cancer murine model. (A)** Schematic diagram of the mouse experiment. When the tumors were palpable, mice were randomly grouped and received intravenous administration of AC_4_ManNA_Z_ (40 mg/kg), Mannose (40 mg/kg) or PBS for 3 consecutive days, DP (5 mg/kg) or PBS was injected via the tail vein on the fourth day right after the administration of AC_4_ManNA_Z_, and this injection procedure was repeated every week. Mice were sacrificed at 28 d after tumor challenge. **(B)** Representative bioluminescence images of BALB/c mice bearing 4T1 tumor allografts (left panel) and the quantification (right panel). **(C)** The tumor curves of the 4T1 tumor allografts. **(D)** Images of the tumor allografts dissected 28 days post-inoculation. **(E)** Tumor weights of the tumor allografts. **(F)** Kaplan-Meier survival curve based on the result of treatment-related death or humane endpoint when the tumors reached ~1000 mm^3^. Data are shown as means±SEM (n = 6 mice, *P < 0.05 by two-way ANOVA test).

The anti-tumor effect of MDP neoantigen was further investigated in mice implanted with 4T1 breast cancer cells that express luciferase (**Figure 4A**). The growth of tumor allografts monitored by live imaging showed that compared with the control groups treated with PBS or noncovalently linked adjuvants, the combination of AC_4_ManNA_Z_ and DP, which was able to covalently link DP on the surface of tumor cells (**Figure 3D** and **Figure S3B**) to generate MDP neoantigen, significantly suppressed the growth of the breast cancer allografts (**Figure 4B**). These results were further confirmed by the tumor growth curves measured by calipers and the weights of dissected allografts 28 days post-implantation (**Figure 4C-E**). Moreover, the *in vivo* generation of the MDP neoantigen by giving AC_4_ManNA_Z_ and DP dramatically increased the survival of the mice bearing breast cancer allografts (**Figure 4F**).

We also tested the toxicity of the *in vivo* generated MDP neoantigen by histochemistry and immunohistochemistry (IHC) analyses of the major vital organs, which showed no pathologic changes (**Figure S4A**) and no inflammatory infiltration in the vital organs (**Figure S4B**). Also, the MDP neoantigen did not affect the bone marrow and peripheral blood (**Figure S4C-D**) as well as the body weight of the mice (**Figure S4E**).

### The *in vivo* generated MDP neoantigen activates the cellular and humoral immune responses against the tumor

**Figure 5:**
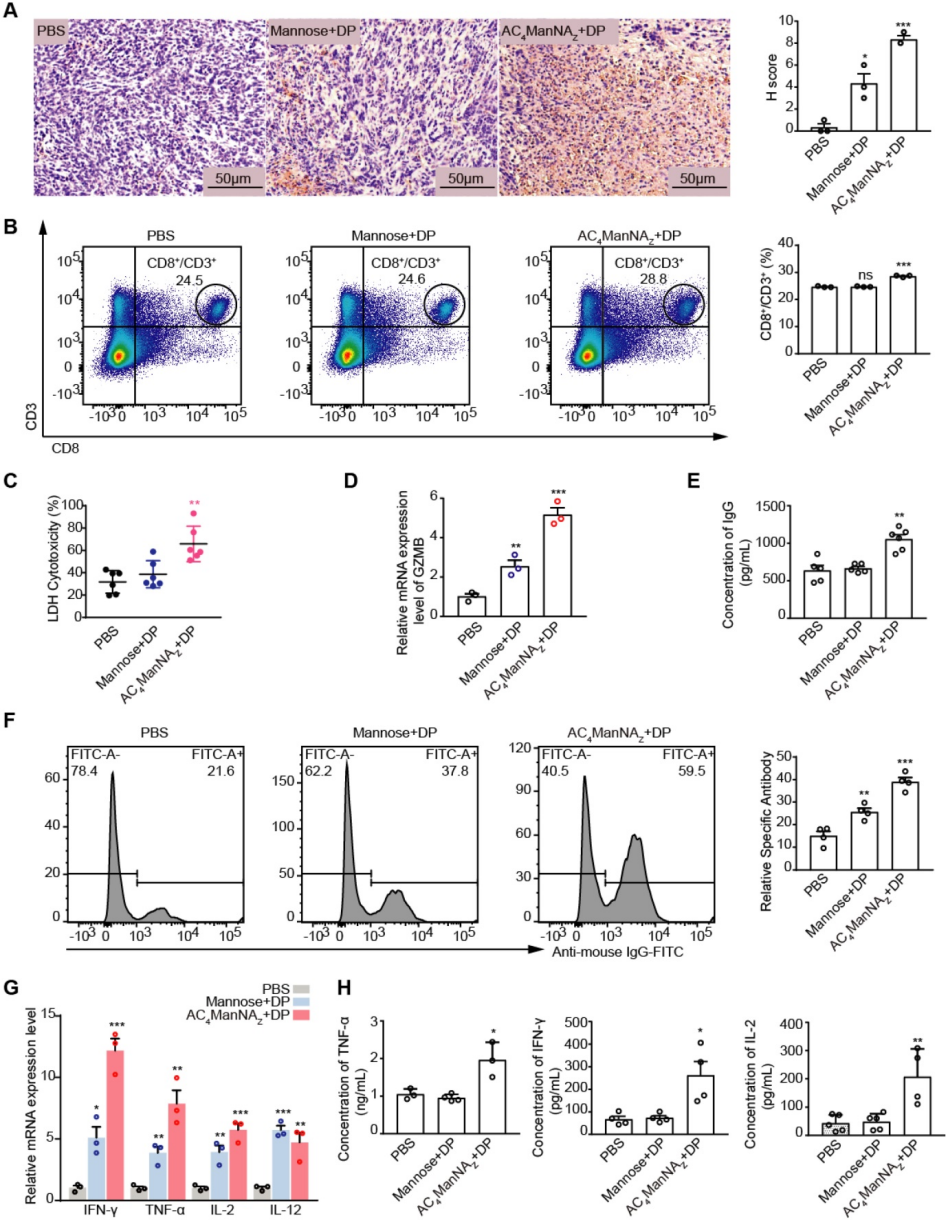
** The MDP neoantigen activated both the cellular and humor immune responses. (A)** Representative images of CD8^+^ T cell infiltration in tumor with anti-CD8α antibody (left panel) and quantification (right panel) by IHC analysis. **(B)** The percentage of CD8^+^ T cells in spleen analyzed by flowcytometry. **(C)** The cytotoxicity of CTL towards 4T1 tumor cells analyzed by LDH assay. **(D)** Analysis of GZMB by qRT-PCR assay. **(E)** Levels of neutralizing antibody (total IgG) analyzed by ELISA. **(F)** The levels of antigen-specific IgG in serum analyzed by flowcytometry. **(G)** Analysis of the indicated pro-inflammatory cytokines by qRT-PCR assay. **(H)** Levels of IL-2, TNF-α and IFN-γ in the serum by ELISA analysis. Data are shown as means±SEM (n = 3-6 mice, ns not significant, *P < 0.05, **P < 0.01, ***P < 0.001 by unpaired Student's t-test).

To investigate the effects of MDP neoantigen on the anti-tumor immune responses, we firstly measured the proportion of infiltration of T cells in the 4T1 breast cancer allografts by IHC staining for CD8 and found the significantly increased CD8+ T cell infiltration in the tumor allografts treated with AC_4_ManNA_Z_ and DP (**Figure 5A**). Increased CD8+ T cell population was also detected in the spleen by the flow cytometry analysis (**Figure 5B**), which showed increased CTL cell activity (**Figure 5C-D**).

The MDP neoantigen was also able to enhance the humoral immune response, which was shown by the increased production of the neutralizing antibody (**Figure 5E**) and the specific antibody (**Figure 5F**). Increased production of the Th1 type of inflammatory cytokines, including IL-2, TNF-α and IFN-γ, were also detected by qRT-PCR (**Figure 5G**) and ELISA (**Figure 5H**) in MDP neoantigen-generated mice.

### MDP neoantigen synergizes with ICIs in the immunotherapy of the breast cancer in mice

Although the generation of MDP neoantigen increased the infiltration of T cells in the breast cancer allografts (**Figure 5A**), the anti-tumor efficacy was not ideal as expected. We suspected that MDP introduction might cause enhanced immune checkpoint mechanism for the evasion of the tumor cells from the immune attack. Therefore, we examined the expression of a major immune checkpoint utilized by tumor cells, i.e., PD-L1, in MDP neoantigen introduced 4T1 allografts. Concurrent with the increased CD8+ T cell infiltration upon MDP neoantigen generation, dramatically enhanced PD-L1 expression was also observed (**Figure S5A-B**), suggesting the combination of MDP neoantigen and ICIs might evoke the strongest anti-tumor immune responses.

**Figure 6:**
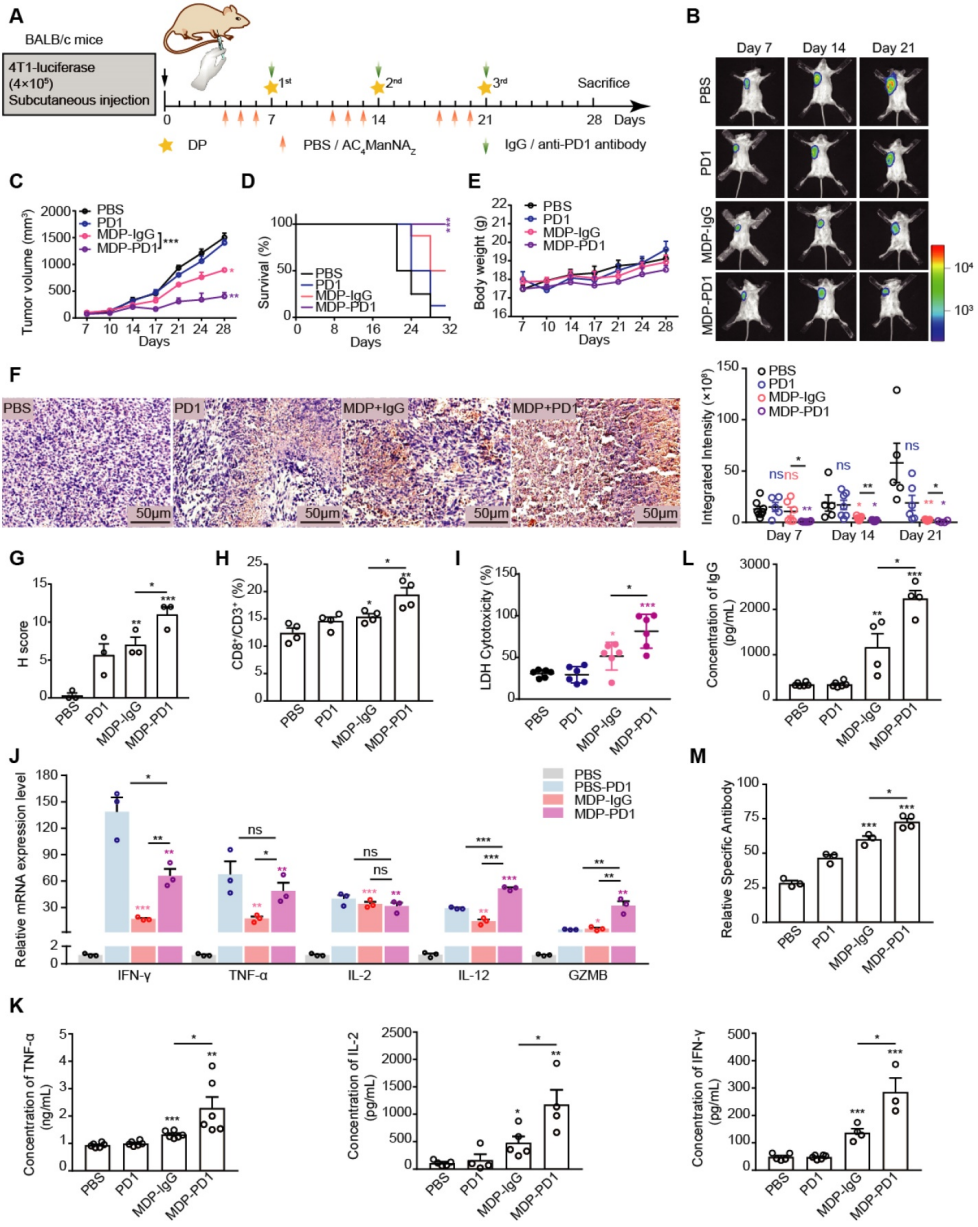
** MDP neoantigen sensitized the breast cancer to the immunotherapy using anti-PD1 therapy. (A)** Schematic diagram of the mouse experiment. When the tumors were palpable, mice were randomly grouped and received intravenous administration of AC_4_ManNA_Z_ (40 mg/kg), Mannose (40 mg/kg) or PBS for 3 consecutive days, DP (5 mg/kg) or PBS and anti-PD1 antibody (10 mg/kg) or anti-IgG-2a antibody (10 mg/kg) were injected via the tail vein on the fourth day right after the administration of AC_4_ManNA_Z_, and this injection procedure was repeated every week. Mice were sacrificed at 28 d after tumor challenge.** (B)** Representative bioluminescence images of BALB/c mice on different time points after inoculation of tumor cells and statistical results of average integrated bioluminescence intensity. **(C)** Statistical results of anti-tumor efficacy study concerning average tumor volume. **(D)** Kaplan-Meier survival curve based on the result of treatment-related death or humane endpoint when the tumors reached ~1000 mm^3^. **(E)** Statistical results of body weight. **(F-G)** Representative images of tumor sections with CD8^+^ T cell infiltration by staining with anti-CD8α antibody (up panel) and quantification of positive IHC presented as H-score (bottom panel). **(H)** The percentage of CD8^+^ T cells in spleen by analyzing with flowcytometry. **(I)** The cytotoxicity of CTL towards 4T1 tumor cells by analyzing with LDH assay. **(J)** Relative mRNA expression level of inflammatory cytokines in tumors from each group of mice analyzed by qRT-PCR. **(K-L)** Level of neutralizing antibody (total IgG) and pro-inflammatory cytokines (IL-2, TNF-α, IFN-γ) in serum from each group of mice by analyzing with ELISA.** (M)** Level of antigen-specific IgG in serum from each group of mice analyzed by flowcytometry. Data are shown as means±SEM (n = 3-6 mice, ns not significant, *P < 0.05, **P < 0.01, ***P < 0.001 by Student's t-test).

To test this hypothesis, we added anti-PD1 antibody treatment right after the *in vivo* generation of MDP neoantigen by the sequential administration of AC_4_ManNA_Z_ and DP in mice inoculated with 4T1 breast cancer allografts (**Figure 6A**). Strikingly, compared with single treatment with anti-PD1 antibody or MDP neoantigen alone, combined treatment dramatically suppressed the growth of the breast cancer (**Figure 6B-C**) and greatly enhanced the survival of tumor-bearing mice (**Figure 6D**), while no toxicity was observed by monitoring the body weight change (**Figure 6E**) and analyzing the peripheral blood haematology and biochemistry (**Table S2**). It was worth notice that 4T1 tumor allografts showed no response to the single treatment of anti-PD1 antibody at all as reported 31. As a comparison, the generation of MDP neoantigen dramatically sensitized this breast cancer to the anti-PD1 antibody, highlighting that the lack of tumor neoantigen accounts for the low response of some types of cancers to ICIs.

We further investigated the immune responses evoked by the combined treatment of anti-PD1 antibody and MDP neoantigen. The combined treatment further enhanced CD8+ T cell infiltration in the tumor (**Figure 6F-G**) and its population in the spleen (**Figure 6H, Figure S6A**) with even stronger killing activity (**Figure 6I**). In addition, the combined treatment was able to further boost the production of the pro-inflammatory factors (**Figure 6J, K**). In addition, both production of the neutralizing antibody and the specific antibody were increased more upon combined treatment (**Figure 6L-M, Figure S6B**).

### MDP neoantigen synergizes with ICIs in the immunotherapy of NSCLC in mice

Given that abnormal cell surface glycosylation is a common feature of many different types of tumor cells 32, we postulated that the *in vivo* generation of MDP neoantigen should be applicable for a broad range of tumors. Therefore, we tested the efficacy of MDP in a NSCLC allograft murine model (**Figure S7A**), which also shows relatively low response to ICIs. As shown in (**Figure S7B**), the single treatment of either MDP neoantigen or anti-PD1 antibody significantly suppressed the growth of NSCLC allografts monitored by live imaging. However, the combinated treatment showed even stronger inhibition on NSCLC growth, which was further confirmed by the histochemistry analysis of the lung sections (**Figure S7C**). In addition, the combined treatment totally prevented the metastasis of NSCLC from the right lung to the left (**Figure S7C**) and dramatically increased the survival of NSCLC-bearing mice (**Figure S7D**), while there was no obvious change of the body weight of the mice, indicating low toxicity of the treatment (**Figure S7E**).

We also observed the activated T-cell-dependent immune responses by the combined treatment of anti-PD1 antibody and MDP neoantigen. Upon the combined treatment, we observed the increased CD8+ T cell population in the spleen (**Figure S7F, Figure S8A**) with even stronger killing activity (**Figure S7I**). In addition, the combined treatment further boosted the production of the pro-inflammatory factors (**Figure S7H, I**). And the production of the neutralizing antibody and the specific antibody were both more increased upon combined treatment (**Figure S7J-K, Figure S8B**).

## Discussion

Tumor neoantigens using the synthetic peptides with mutations identified by genome sequencing in cancer patients have been demonstrated to be an efficient strategy to evoke the immune responses against cancer cells 33. However, the preparation of this sort of tumor neoantigens is quite expensive and time-consuming. In this study, we took the advantage of the tumor-associated carbohydrate antigens (TACAs), which are broadly present on the cell surface of malignantly transformed cells and can invoke a T-cell-independent humoral response 34, and the pattern molecular adjuvant Pam3CSK4, which can bind TLR1/2 to activate the innate immune system, in order to eventually elicit a T-cell dependent immune response. By utilization of a sialic acid glycoengineering method with AC_4_ManNA_Z_, we successfully linked DBCO-conjugated Pam3CSK4 to the cell surface carbohydrate groups covalently through the click chemical reaction to generate the artificial tumor neoantigen MDP both in cultured cancer cells and in tumor allografts in mice. And the generation of MDP neoantigen in tumor cells efficiently activated both the humoral and the T-cell-dependent immune responses that strongly suppressed the tumor growth and improved the survival of tumor-bearing mice. More importantly, for the cancer types that showed no or very low responses to immune therapy with ICIs alone, the *in vivo* generation of MDP neoantigen greatly sensitized these cancer types to ICIs and achieved dramatically enhanced anti-tumor outcome.

Chemically synthesized azido saccharides are inactive derivatives of natural monosaccharide, which can be integrated into intracellular proteins through the post-transcriptional glycosylation process and expressed on the cell surface. It has the advantages of low background noise, unlimited reaction conditions (temperature / PH value), high specificity, and almost no toxic by-products 35. And Pam3CSK4 is an innate immune system agonist, which was mainly used as an anti-tumor adjuvant by physically mixed 36 or chemically combined 37 with other anti-tumor therapeutic drugs to stimulate the anti-tumor immune responses. Here, to further improve the antigenicity and immunogenicity, we combined these two groups on the surface of cancer cells to generate the complex MDP as a powerful neoantigen, which was able to activate the antigen presentation function of the DCs of innate immune system and eventually activate the naive T lymphocytes and the adaptive immune system. Therefore, in this study, Pam3CSK4 functions not only as an adjuvant but also as an antigen.

In this study, mannose was set as a negative control of AC_4_ManNA_Z_ due to the lack of azide group (-N3), which allowed us to evaluate the effects of unconjugated 'DP' *in vivo*. The tetra-acetylated ManNAc which contains an acetyl group should be a more rigorous control.

When administrated in mice, AC_4_ManNA_Z_ was able to be uptaken by relatively broad range of organs besides the tumor, including the liver and the kidney when tracked by small DBCO-Cy5 molecule that could easily enter the cells. As both the liver and kidney are blood-rich organs, we hypothesize that the DBCO-Cy5-conjugated glycoproteins in the blood may cause the accumulation of DBCO-Cy5 in those vital organs. Therefore, we performed cardiac perfusion before the tracing experiment by IVIS fluorescence imaging. Only tumor cells showed very active glycosylation that introduced AC_4_ManNA_Z_ into sialic acid synthesis pathway and presented the azide group on the cell surface to ensure the specifically covalent linkage of a larger DP group on the surface of tumor cells when tracked by the larger DP-Rhodamine molecule that cannot enter the cells. This feature, together with the low toxicity of chemically synthesized AC_4_ManNA_Z_ and the high specificity of the click chemical reaction, contributes the low toxicity of the generation of MDP in mice. To ensure the highest safety for the clinical therapy in future, delivery systems based on antibody-conjugated nanoparticles might be used to specifically deliver AC_4_ManNA_Z_ into the tumor cells.

The application of ICIs, such as CTLA-4 and PD1/PD-L1 antibodies, has revolutionized the immune therapy of cancers. However, limited efficacy of ICIs has been observed in the clinical treatment of various cancer types. The dependence of ICI clinical outcomes on the mutation load of tumor cells strongly indicates that the lack of enough tumor neoantigen dampens the effect of ICIs. Although the MDP neoantigen can robustly increase the population of CD8+ T cells, the tumor cells may strengthen the immune checkpoint mechanism to evade the immune attack. We did observe the increased PD-L1 level in the tumor allografts upon MDP generation, highlighting the importance of the combination of increasing tumor neoantigen and ICIs for achieving the best immune therapy outcome.

## Conclusions

Here, we performed *in vivo* generation of the tumor neoantigen by using a sialic acid glycoengineering method with AC_4_ManNA_Z_ and DBCO-conjugated Pam3CSK4 through the click chemical reaction which can efficiently activate both the humoral and the T-cell-dependent immune responses to suppress the tumor growth and improve the survival of tumor-bearing mice. More importantly, the *in vivo* generation of MDP neoantigen can greatly sensitize a broad range of cancer types to ICIs treatment and dramatically improve the anti-tumor efficacy of current immune therapy using ICIs.

## Supplementary Material

Supplementary figures and tables.

## Abbreviations

DP: DBCO-Pam3CSK4
ICIs: immune checkpoint inhibitors
AC_4_ManNA_Z_: tetra acetyl-N-azidoacetylmannosamine
MDP: ManNA_Z_-EBCO-Pam3CSK4
NSCLC: non-small cell lung cancer
CTLs: cytotoxic T cells
TACAs: tumor-associated carbohydrate antigens
DCs: dendritic cells
PRRs: pattern recognition receptors
TLR: toll-like receptors
