# Supplementary material for: Generation of triacyl lipopeptide-modified glycoproteins by metabolic glycoengineering as the neoantigen to boost anti-tumor immune response
Source: Theranostics. 2021 May 25;11(15):7425–38. doi: 10.7150/thno.60211 (PMC8210591; doi:10.7150/thno.60211)
Supplement: Supplementary file 1 — Supplementary figures and tables. [file thnov11p7425s1.pdf]

Supplementary Figures and legends

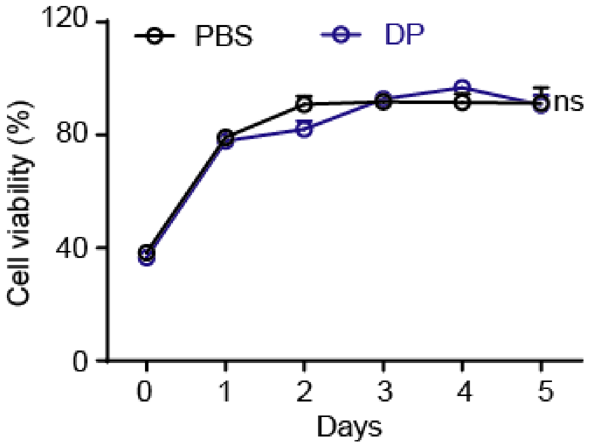

Figure S1. CCK8 results of DP in 4T1 breast cancer cell line *in vitro*. Statistical results of cell viability from different groups. Data are shown as means ± SD (n = 3, ns not significant by Student's t-test).

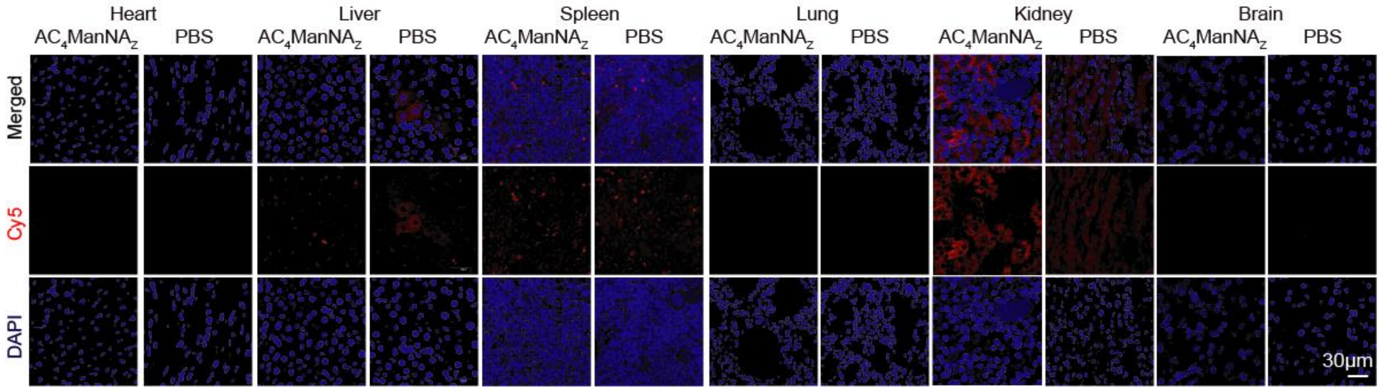

Figure S2. IF results of Fatal organs in cancer-selective labeling *in vitro*. CLSM images of representative immunofluorescence of fatal organ sections from different groups by IF staining.

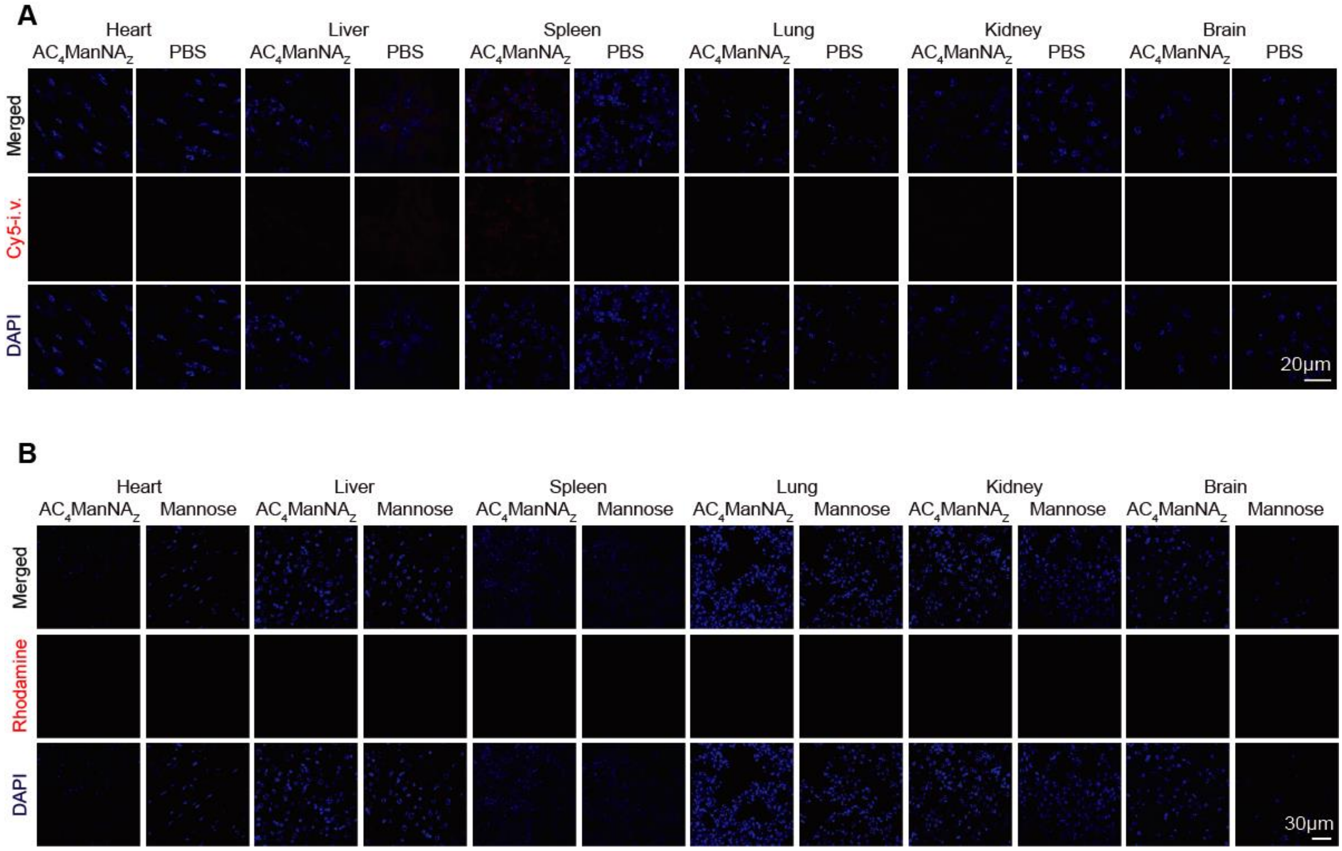

Figure S3. IF results of Fatal organs in cancer-selective labeling *in vivo.* **(A)** CLSM images of representative immunofluorescence of fatal organ sections from different groups treated with i.v. DBCO-Cy5 by IF staining. **(B)** CLSM images of representative immunofluorescence of fatal organ sections from different groups treated with i.v. DP-Rhodamine by IF staining.

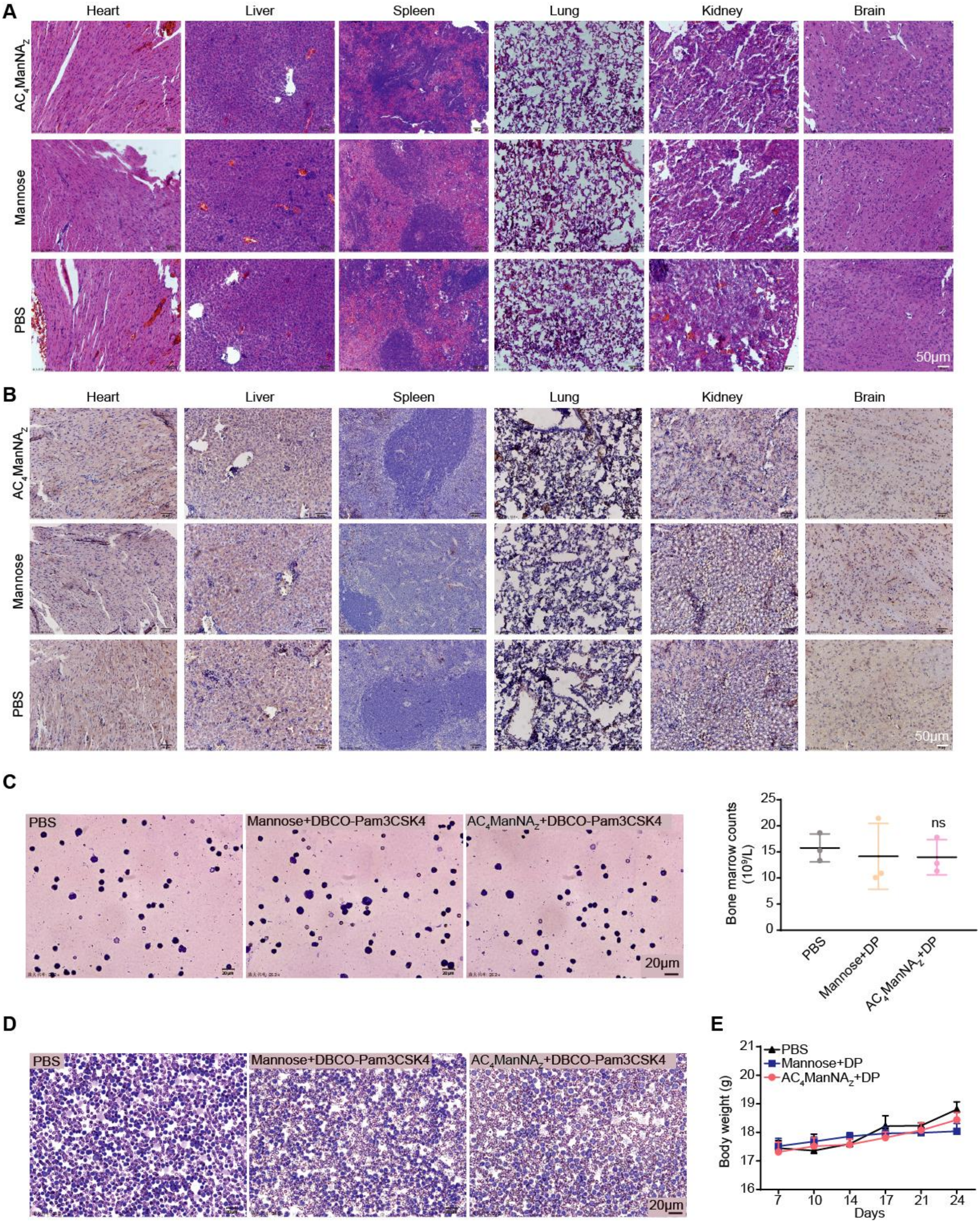

Figure S4. The toxicity of mouse in different groups. (A-B) Representative images of fatal organs by staining with H&E **(A)** and anti-CD8α antibody **(B)**. **(C)** Representative images of bone marrow smear by staining with Giemsa (left panel) and the statistical results of bone marrow counts (right panel). **(D)** Representative mages of peripheral blood smear by staining with Wright. **(E)** Statistical results of body weight. Data are shown as means ± SEM (n = 3-6 mice, ns not significant by Student's t-test).

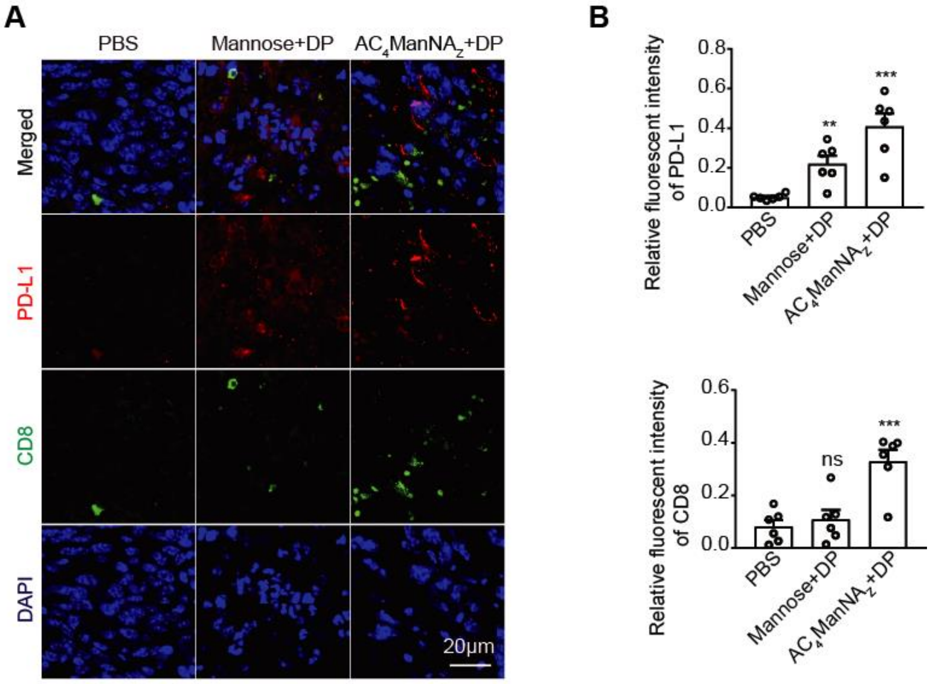

Figure S5. IF co-staining results of PD-L1 and CD8 in tumor sections. (A) Representative images of the CD8 and PD-L1 signals in the tumor allografts by IF staining. **(B)** Average fluorescence intensity of CD8 and PD-L1 in tumor sections described in **(A)**. Data are shown as means±SEM (n = 6 mice, ns not significant, **P < 0.01, ***P < 0.001 by Student's t-test).

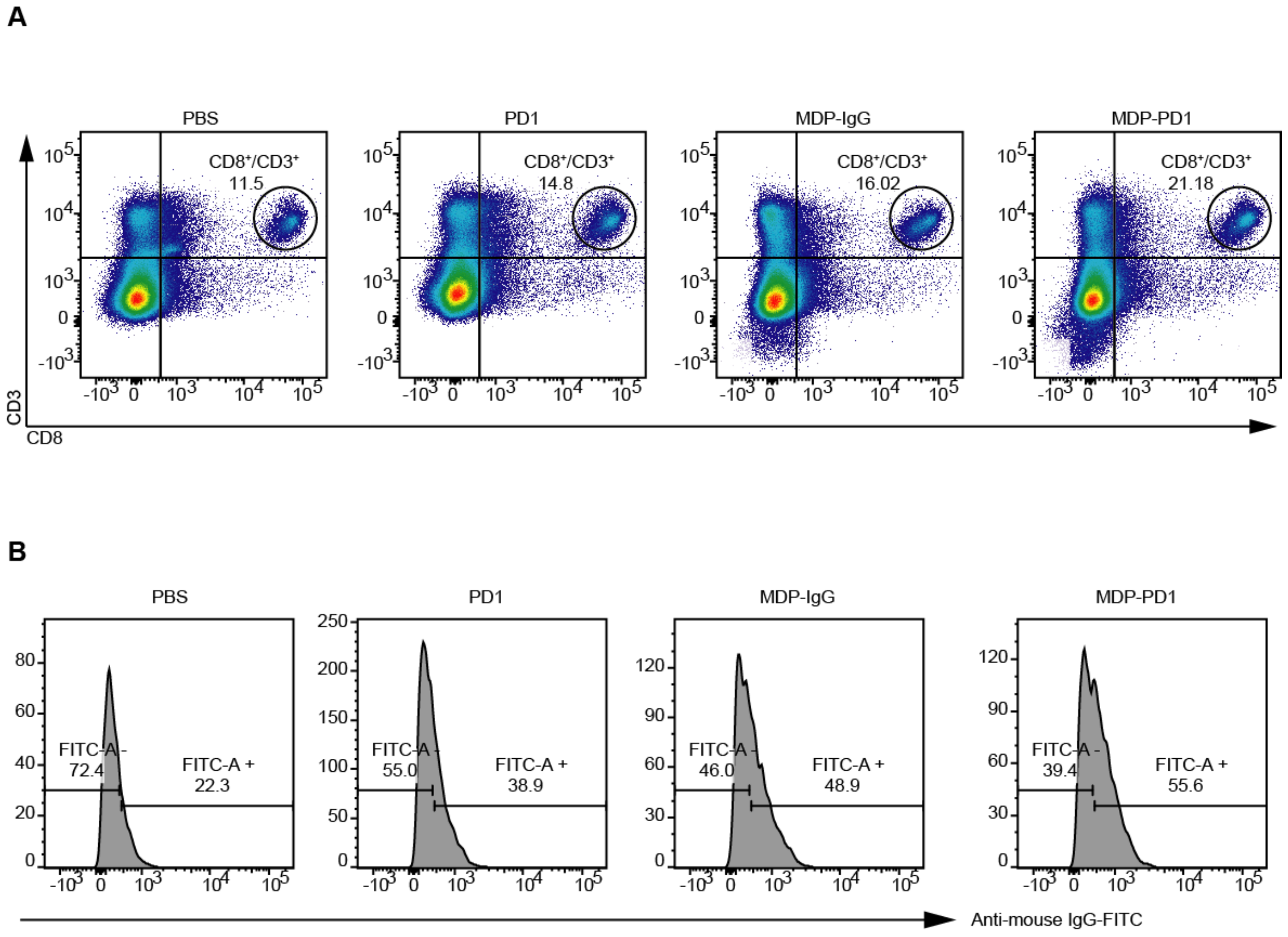

Figure S6. The original FACs result in breast cancer mouse model. (A) The original FACs result of the percentage of $CD8^+$ T cells in spleen. **(B)** The original FACs result of antigen-specific IgG in serum.

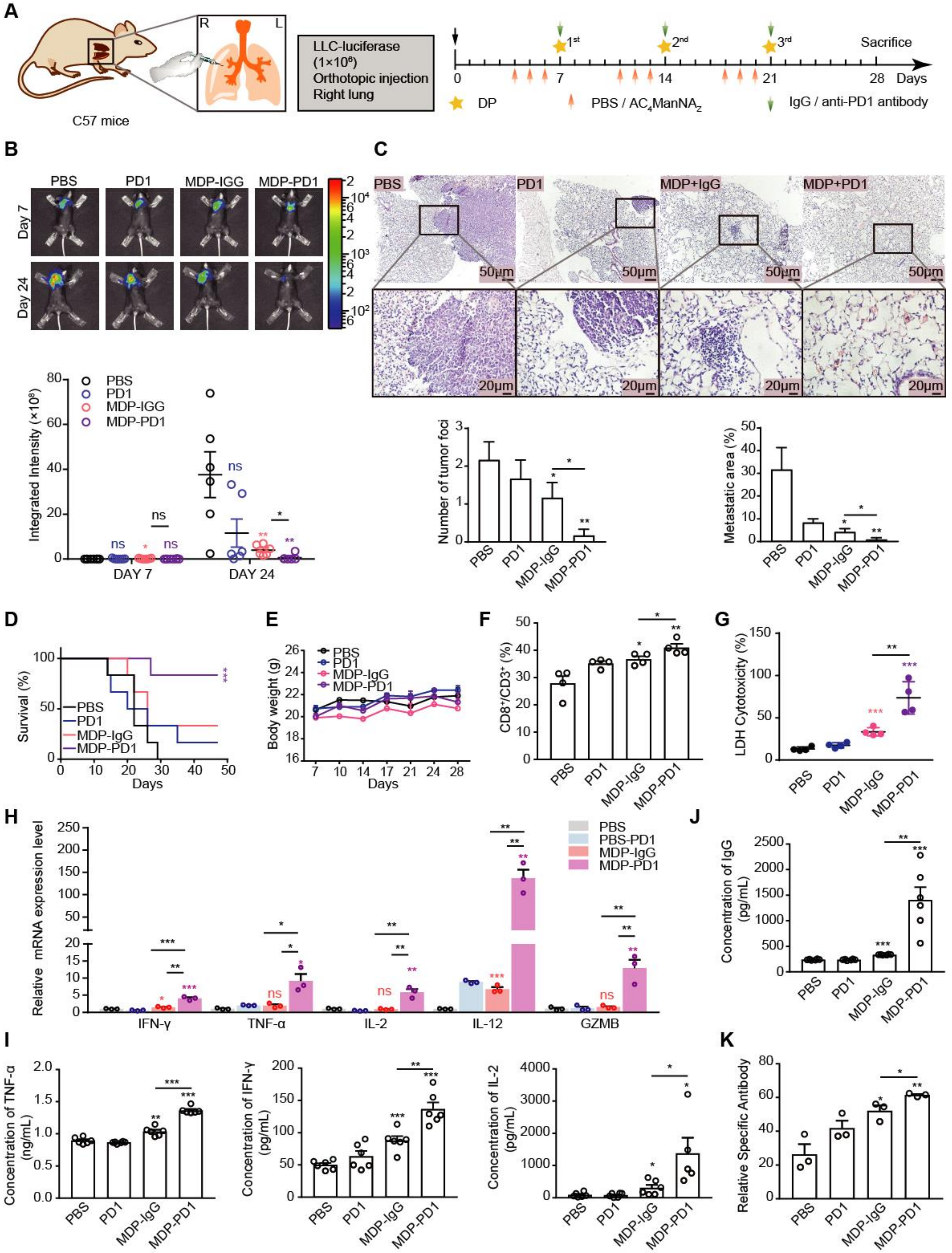

A
C57 mice
R
L
LLC-luciferase
(1×10⁶)
Orthotopic injection
Right lung
1st
2nd
3rd
Sacrifice
0
7
14
21
28
Days
DP
PBS / AC4ManNAz
IgG / anti-PD1 antibody
B
PBS
PD1
MDP-IGG
MDP-PD1
Day 7
Day 24
Integrated Intensity (×10⁸)
DAY 7
DAY 24
ns
C
PBS
PD1
MDP+IgG
MDP+PD1
50μm
20μm
Number of tumor foci
Metastatic area (%)
MDP-IgG
D
Survival (%)
Days
E
Body weight (g)
Days
F
CD8⁺/CD3⁺ (%)
G
LDH Cytotoxicity (%)
H
Relative mRNA expression level
PBS-PD1
IFN-γ
TNF-α
IL-2
IL-12
GZMB
J
Concentration of IgG (pg/mL)
I
Concentration of TNF-α (ng/mL)
Concentration of IFN-γ (pg/mL)
Concentration of IL-2 (pg/mL)
K
Relative Specific Antibody

Figure S7. MDP neoantigen improved the antitumor efficacy of the anti-PD1 antibody in treating NSCLC in mice. (A) Schematic diagram of the experimental mouse model design. When the tumors were palpable, mice were randomly grouped and received intravenous administration of AC_4ManNA_Z (40 mg/kg), Mannose (40 mg/kg) or PBS for 3 consecutive days, DP (5 mg/kg) or PBS and anti-PD1 antibody (10 mg/kg) or anti-IgG-2a antibody (10 mg/kg) were injected via the tail vein on the fourth day right after the administration of AC_4ManNA_Z, and this injection procedure was repeated every week. Mice were sacrificed at 28 d after tumor challenge. **(B)** Representative bioluminescence images of C57BL/6 mice on different time points after inoculation of tumor cells and statistical results of average integrated bioluminescence intensity. **(C)** Representative images of tumor sections by staining with H&E (up panel) and statistical results of numbers and area percentages of in situ and metastatic tumor lesions (bottom panel)**. (D)** Kaplan-Meier survival curve based on the result of day 47. **(E)** Statistical results of body weight. **(F)** The percentage of $CD8^+$ T cells in spleen analyzed by flowcytometry. **(G)** The cytotoxicity of CTL towards LLC tumor cells by analyzing with LDH assay. **(H)** Relative mRNA expression level of inflammatory cytokines in tumors from each group of mice analyzed by qRT-PCR. **(I-J)** Level of neutralizing antibody (total IgG) and pro-inflammatory cytokines (IL-2, TNF-α, IFN-γ) in serum by analyzing with ELISA. **(K)** Level of antigen-specific IgG in serum analyzed by flowcytometry. Data are shown as means ± SEM (n = 3-6 mice, ns not significant, *P < 0.05, **P < 0.01, ***P < 0.001 by Student's t-test).

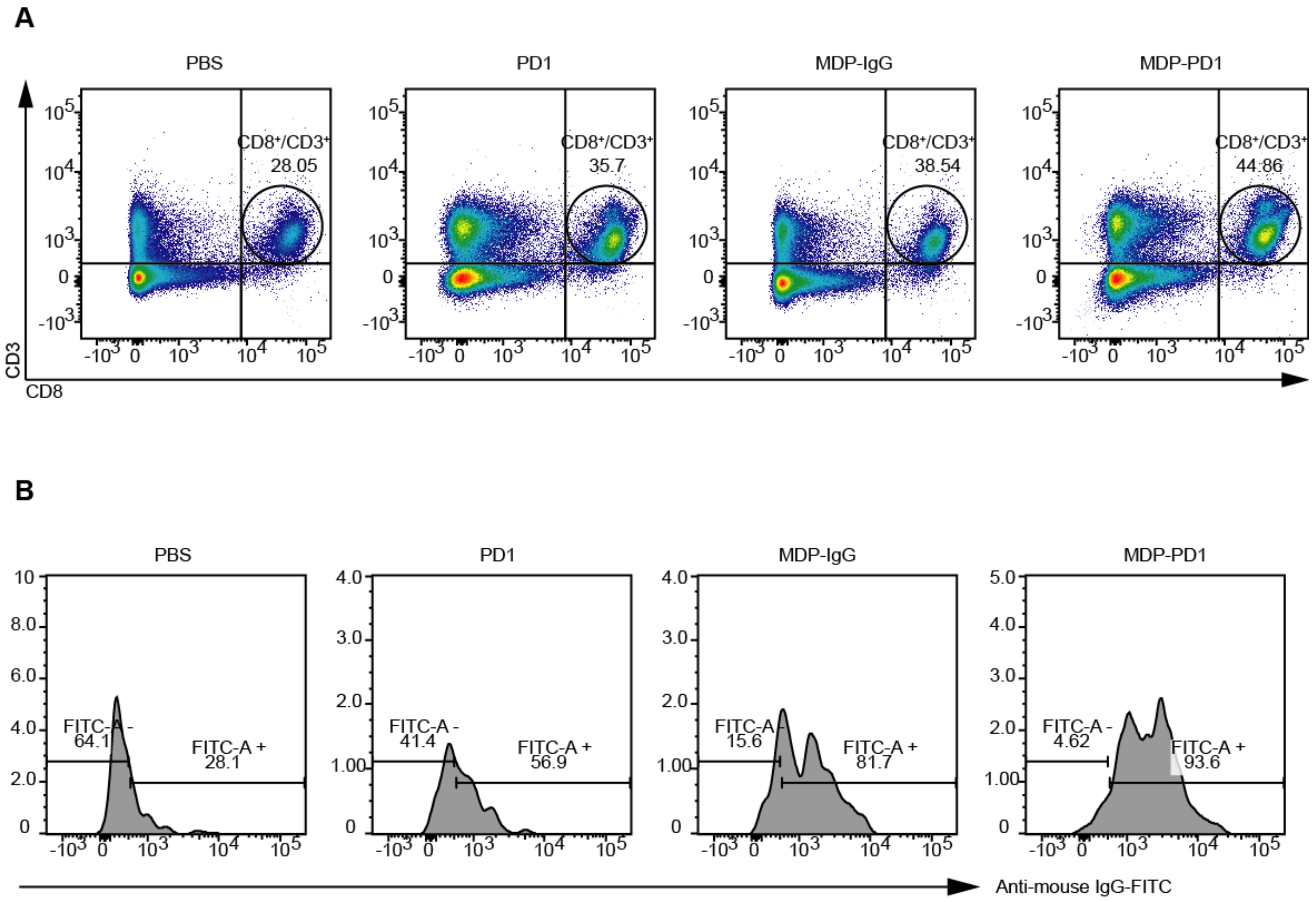

Figure S8. The original FACs result in lung cancer mouse model. (A) The original FACs result of the percentage of $CD8^+$ T cells in spleen. **(B)** The original FACs result of antigen-specific IgG in serum.

Table S1.

Primers for quantitative RT–PCR.		
**Primer**		**Sequences (5’-3’)**
Mouse CD8a	(Forward)	CAAATGTCCCAGGCCGCTA
	(Reverse)	TTCCTGGCGGTGCCATTTTA
Mouse IL-6	(Forward)	GTCCTTCCTACCCCAATTTCCA
	(Reverse)	TAACGCACTAGGTTTGCCGA
Mouse IL-2	(Forward)	GCCCCAAGGGCTCAAAAATG
	(Reverse)	GCGCTTACTTTGTGCTGTCC
Mouse IL-12	(Forward)	TCTTCTCACCGTGCACATCC
	(Reverse)	TGGCCAAACTGAGGTGGTTT
Mouse IFN-γ	(Forward)	GAGGTCAACAACCCACAGGT
	(Reverse)	GGGACAATCTCTTCCCCACC
Mouse ACTB	(Forward)	TGAGCTGCGTTTTACACCCT
	(Reverse)	GCCTTCACCGTTCCAGTTTT
Mouse TNF-α	(Forward)	ATGGCCTCCCTCTCATCAGT
	(Reverse)	TTTGCTACGACGTGGGCTAC
Mouse IL-10	(Forward)	GCTCTTGCACTACCAAAGCC
	(Reverse)	CTGCTGATCCTCATGCCAGT
GZMB	(Forward)	GGACAAAGGCAGGGGAGATCA
	(Reverse)	AAGGAAGCCCCCACATATCG

Table S2.

Effects of MDP on haematological and blood biochemical results in mice.

Parameters	Control	PD1	MDP-IgG	MDP-PD1
WBC (E^3/ul)	532.4±169.9	623.1±207.9	651.4±216.5	472.3±173.8
EO (%)	3.2±1.3	3.6±1.82	1.4±0.89	2.6±1.14
BA (%)	0.66±0.114	0.78±0.239	0.84±0.288	0.68±0.13
RBC (E^6/ul)	8.588±0.5	6.666±1.9	7.766±0.5	8.242±0.4
HCT (%)	39.54±1.965	31.1±7.675	36.18±2.132	36.8±1.661
MCV (fL)	46.06±0.68	47.04±1.816	46.64±0.893	44.68±0.65
MCH (pg)	15.06±0.391	15.06±0.261	15.32±0.415	14.8±0.158
MCHC (g/L)	326.8±5.76	320.4±11.91	327.8±7.05	330.8±5.81
RDW-CV (%)	25.44±0.844	239±13.62	248.8±5.45	245.6±2.88
PLT (E^3/ul)	1170.4±299.9	767±306.9	1068.8±10.6	1301.8±144.8
MPV (fL)	8±0.141	8.14±0.251	7.96±0.195	8.1±0.308
ALT (U/L)	24.5±9.19	25±2.83	25.5±4.95	27.67±5.13
AST (U/L)	257±96.17	226.5±34.65	415±19.8	303.33±154.78
TP (g/L)	53.25±0.07	57.7±3.39	47.75±1.63	57.13±4.15
ALB (g/L)	31.7±0.28	33.1±0.42	24.6±5.94	28.4±9.26
BUN (mmol/L)	7.55±0.92	6.3±0.71	9.1±2.55	6.2±1.54
CRE (μmol/L)	46±8.49	38.5±2.12	46±18.38	84±67.1

Notes: All values are shown as the means ± SD. (n = 3 in each group). WBC, white blood cell; RBC, red blood cell; MCV, mean corpuscular volume; MCH: mean corpuscular hemoglobin; MCHC: mean corpuscular hemoglobin concentration; MPV, mean platelet volume; PLT, blood platelet; RDW-CV, red blood cell distribution width variation coefficient; ALT, Alanine aminotransferase; AST, Aspartate aminotransferase; TP, total serum protein; ALB, serum albumin; BUN, blood urea nitrogen; CRE, creatinine.
